# Fine-tuned large Language model for extracting newly identified acute brain infarcts based on computed tomography or magnetic resonance imaging reports

**DOI:** 10.1007/s10140-025-02354-1

**Published:** 2025-06-02

**Authors:** Nana Fujita, Koichiro Yasaka, Shigeru Kiryu, Osamu Abe

**Affiliations:** 1 Department of Radiology, National Center for Global Health and Medicine, Japan Institute for Health Security, Tokyo, Japan; 2 Department of Radiology, The University of Tokyo, Tokyo, Japan; 3 Department of Radiology, International University of Health and Welfare, Narita, Japan

**Keywords:** Brain infarction, Artificial intelligence, Natural language processing, Large language model, Magnetic resonance imaging, Computed tomography

## Abstract

**Purpose:**

This study aimed to develop an automated early warning system using a large language model (LLM) to identify acute to subacute brain infarction from free-text computed tomography (CT) or magnetic resonance imaging (MRI) radiology reports.

**Methods:**

In this retrospective study, 5,573, 1,883, and 834 patients were included in the training (mean age, 67.5 ± 17.2 years; 2,831 males), validation (mean age, 61.5 ± 18.3 years; 994 males), and test (mean age, 66.5 ± 16.1 years; 488 males) datasets. An LLM (Japanese Bidirectional Encoder Representations from Transformers model) was fine-tuned to classify the CT and MRI reports into three groups (group 0, newly identified acute to subacute infarction; group 1, known acute to subacute infarction or old infarction; group 2, without infarction). The training and validation processes were repeated 15 times, and the best-performing model on the validation dataset was selected to further evaluate its performance on the test dataset.

**Results:**

The best fine-tuned model exhibited sensitivities of 0.891, 0.905, and 0.959 for groups 0, 1, and 2, respectively, in the test dataset. The macrosensitivity (the average of sensitivity for all groups) and accuracy were 0.918 and 0.923, respectively. The model’s performance in extracting newly identified acute brain infarcts was high, with an area under the receiver operating characteristic curve of 0.979 (95% confidence interval, 0.956–1.000). The average prediction time was 0.115 ± 0.037 s per patient.

**Conclusion:**

A fine-tuned LLM could extract newly identified acute to subacute brain infarcts based on CT or MRI findings with high performance.

## Introduction

Stroke is the fifth leading cause of death in the United States [1] and the second leading cause of death worldwide [2]. Furthermore, stroke is the leading cause of acquired long-term disability [2]. Specifically, cerebral infarction is a common form of stroke. The treatment of acute cerebral infarction has advanced significantly in recent years [3], with the establishment of intravenous thrombolysis and endovascular therapy for occluded cerebral arteries. However, some patients are ineligible for these therapies, or do not achieve functional improvement despite receiving treatment. Recognition of subacute brain infarcts is also important. The combination of aspirin and clopidogrel during the subacute phase of stroke is known to be effective in preventing recurrence without increasing the risk of intracerebral hemorrhage or major bleeding [4]. Approximately 75% of patients experience some degree of disability [5]. Patients may experience severe functional impairment that has a lasting impact on their activities of daily living and quality of life. Stroke also has serious consequences for society and the patient’s family [6]. Imaging modalities play a critical role in the management of stroke.

Magnetic resonance imaging (MRI) is the standard modality for diagnosing cerebral infarction due to its high sensitivity, especially for detecting acute infarcts. Although less sensitive, computed tomography (CT) can also visualize acute lesions and remains useful in clinical practice. Detection of both acute and subacute brain infarcts helps in the appropriate management of patients [4]. However, in some cases, these radiological examinations and reports are not adequately reviewed [7], which may delay the recognition of the patient’s impending condition. Automated systems that extract acute-to-subacute cerebral infarction reports can serve as a warning mechanism, preventing clinicians from overlooking unexpected strokes and facilitate rapid assessment. However, the free-text nature of radiology reports makes automated analysis difficult [8].

Natural language processing (NLP), a branch of artificial intelligence, enables computers to understand and interact with human language. Fine-tuned large language models (LLMs) based on BERT have advanced clinical NLP, including applications in radiology [9] with promising potential in various tasks [10–15]. Fine-tuning BERT on radiology-specific datasets improves its performance in radiology-related tasks, such as report classification. Previous studies have reported that LLMs can efficiently extract and classify target patients from medical image management and processing systems with high accuracy and speed for various conditions, including pretreatment lung cancer [16], brain tumors [17], and progressive bone metastases [18]. However, no study has investigated the use of LLMs to classify ischemic stroke, distinguishing between acute to subacute and chronic phases, from unstructured radiology reports.

This study evaluated the performance of a fine-tuned LLM in classifying patients with newly identified acute to subacute ischemic stroke, those with chronic ischemic stroke, and nonstroke cases based on CT or MRI findings from unstructured radiology reports. By exploiting the ability of LLMs to quickly and accurately extract relevant patient information from large datasets, this approach can significantly improve stroke management efficiency and timeliness.

## Materials and methods

Our Institutional Review Board approved this retrospective study, which waived the requirement for written informed consent considering the retrospective nature of this study.

### Datasets

**Fig. 1:**
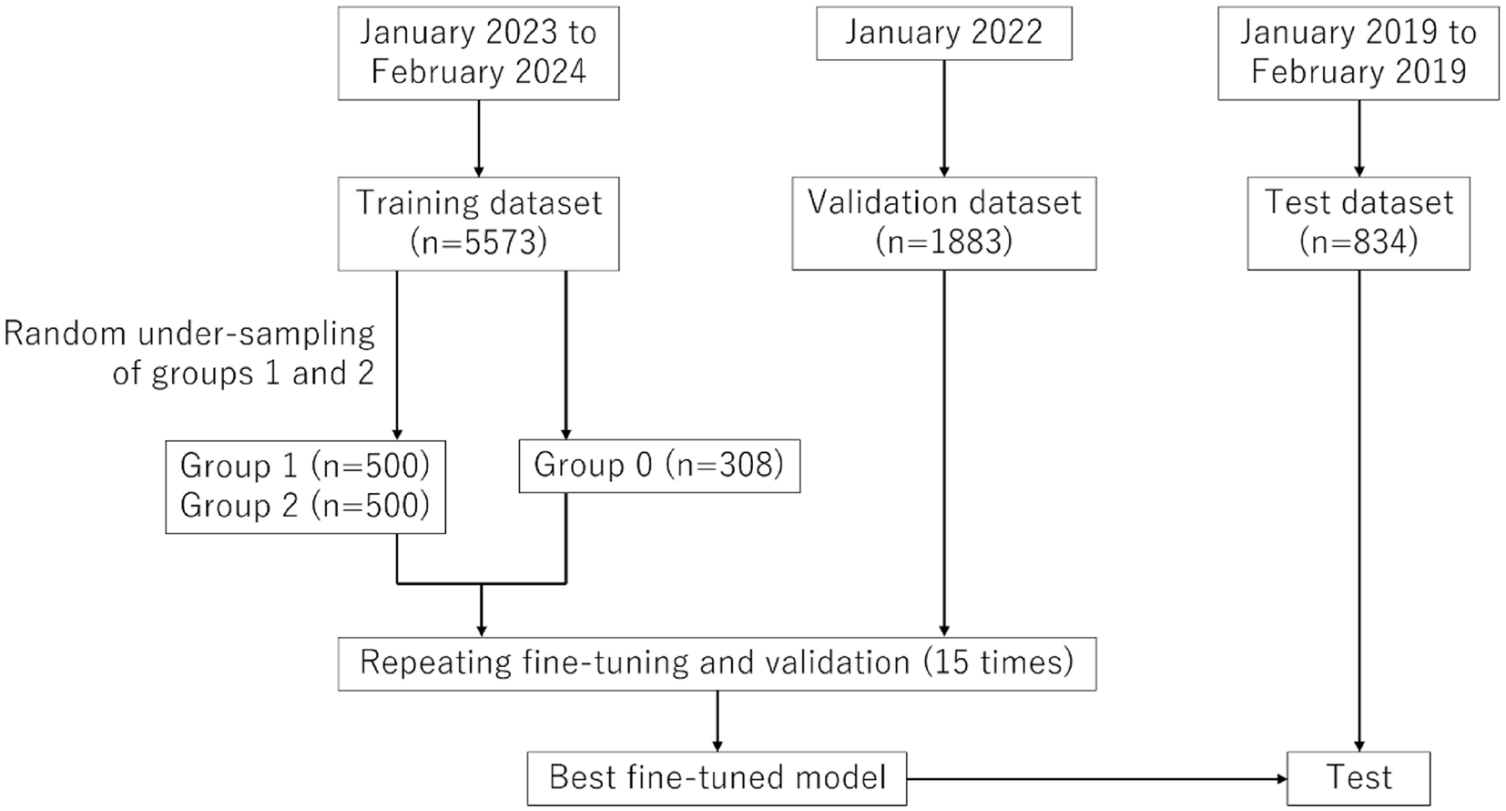
Schematic of the process of data extraction, training, and performance evaluation of the large language model

We searched the medical image management and processing systems of our hospital for eligible cases (Fig. 1). Among patients who underwent head CT or MRI during three time periods—January 2023 to February 2024, January 2022, and January to February 2019—reports containing the keyword “cerebral infarction” in either the clinical indication or imaging diagnosis fields were extracted and saved in CSV format. They were designated as the training, validation, and test datasets, respectively. The number of patients included in each dataset was 5,573, 1,883, and 834, respectively. To enhance the robustness of the results, the training, validation, and test datasets were designed to use data from different time periods. This allowed us to evaluate the trained models on datasets external to time. All reports were written mainly in Japanese by radiologists with ≥ 5 years of imaging experience.

### Undersampling of training datasets

To address class imbalance in the training dataset (308, 2,359, and 2,906 patients in groups 0, 1, and 2, respectively), we randomly undersampled groups 1 and 2 to 500 patients each (Fig. 1). This was done to prevent model bias toward the majority classes and improve overall classification performance.

### Reference standard

The clinical indication and imaging diagnosis sections of the radiology reports were reviewed, and the reports were classified into three groups: group 0, patients with newly identified acute to subacute infarction; group 1, patients with known existing acute to subacute infarction or old infarction; and group 2, patients without infarction. These assessments were performed by a radiologist with 7 years of imaging experience under the supervision of a senior radiologist (14 years of imaging experience).

### Fine-tuning of the pretrained LLM

We used a Japanese language model called BERT (version 3) (https://huggingface.co/cl-tohoku/bert-base-japanese), developed by Tohoku University and pretrained using Japanese Wikipedia articles available up to September 1, 2019. The model works through 12 processing layers - these are like steps in its thinking process, allowing it to gradually build a deeper understanding of the text. It also uses 12 “attention heads” in each layer, which help the model focus on different parts of a sentence at the same time and capture context more accurately. In total, the model has approximately 110 million parameters—adjustable settings that it fine-tunes during training to improve language understanding. To process Japanese text, the model uses a method called subword tokenization, which breaks sentences into smaller parts of words. This is especially useful in Japanese, where words are not separated by spaces, and it allows the model to recognize and understand even rare or unfamiliar terms by analyzing their components. Fine-tuning (custom training) of the pretrained model was performed on a workstation equipped with an Intel Core i9-9900 K processor, NVIDIA Quadro P5000 graphics card, and 64 GB of random-access memory, using Python (version 3.10.13) (https://www.python.org/) and the Hugging Face Transformers library (version 4.35.2) (https://huggingface.co/). The model was adapted for our specific classification task using a built-in method from the library designed for categorizing text (AutoModelForSequenceClassification). It was trained to assign each report to one of three predefined categories. For each report, the model generated a set of numerical scores (logits), one for each category, and the category with the highest score was selected as the predicted outcome. The training session was repeated 15 times with the same hyperparameters and training data to account for the inherent randomness in the fine-tuning process. Based on the model’s performance on the training and validation datasets, the number of epochs (10 epochs) for fine-tuning was determined empirically. The other hyperparameters were set to default values from the Transformers library. In each session, the model was fine-tuned on the training dataset, and its performance was assessed on the validation dataset. To enhance the model’s generalizability, CT and MRI reports were used in these processes. The code used for fine-tuning is available upon reasonable request.

### Test phase of fine-tuned LLM

The performance of the fine-tuned mode, which exhibited the highest macrosensitivity (average of sensitivity for all groups) plus accuracy, was further evaluated using the time-independent test dataset (Fig. 1). The sensitivity, macrosensitivity, accuracy, and time required for prediction were recorded.

### Statistical analysis

Statistical analyses were performed using R (version 4.1.2; https://www.r-project.org/). The performance of the fine-tuned model in differentiating group 0 from groups 1 and 2 was evaluated using receiver operating characteristic curve analysis, and the area under the ROC curve (AUC) was calculated. In this analysis, the model’s output value for group 0 was used. The sensitivity for each group and the accuracy of CT were compared with those of MRI using Fisher’s exact test. The macrosensitivity of CT was compared with that of MRI using the paired *t*-test. *P-*values < 0.050 were used to denote statistical significance.

## Results

### Datasets

Table 1 presents patient background information. In the training dataset, the numbers of patients in groups 0, 1, and 2 were 308, 2,359, and 2,906, respectively. In the validation dataset, the numbers were 31, 259, and 1,503, respectively. In the test dataset, the numbers were 55, 486, and 293, respectively. The mean ages of the patients were 67.5 ± 17.2, 61.5 ± 18.3, and 66.5 ± 16.1 years in the training, validation, and test datasets, respectively. The sex distribution was 2,831 males/2,742 females, 994 males/889 females, and 488 males/346 females in the training, validation, and test datasets, respectively.

**Table 1:** Patient background information and group distribution of each dataset

	Training	Validation	Test
Age (mean ± standard deviation) (years)	67.5 ± 17.2	61.5 ± 18.3	66.5 ± 16.1
Sex (male/female)	2831/2742	994/889	488/346
Number of reports	5,573	1,883	834
Modality			
CT	2,321	828	413
MRI	3,252	1,055	421
Number of patients in each group			
Group 0	308	31	55
Group 1	2,359	259	486
Group 2	2,906	1,503	293

### Training and validation for model selection

Table 2 summarizes the performance of the models in each training session on the validation dataset. The median macrosensitivity and accuracy among the 15 sessions were 0.878 and 0.841, respectively. For further performance evaluation on the test dataset, the model from the 14th training session, which exhibited the highest macrosensitivity (0.916) and accuracy (0.844), was selected as the final model.

**Table 2:** Model performance on the validation dataset

	Sensitivity				Accuracy
Model	Group 0	Group 1	Group 2	Macrosensitivity	
1	0.903	0.884	0.820	0.869	0.831
2	0.968	0.915	0.825	0.903	0.840
3	0.968	0.853	0.837	0.886	0.841
4	1.000	0.896	0.831	0.909	0.842
5	0.871	0.714	0.861	0.816	0.841
6	0.968	0.834	0.842	0.881	0.843
7	1.000	0.873	0.834	0.902	0.842
8	0.935	0.822	0.841	0.866	0.840
9	0.871	0.606	0.878	0.785	0.840
10	1.000	0.807	0.842	0.883	0.840
11	0.871	0.587	0.883	0.780	0.842
12	0.935	0.865	0.833	0.878	0.839
13	0.935	0.768	0.851	0.851	0.841
14	1.000	0.919	0.829	0.916	0.844
15	0.935	0.857	0.836	0.876	0.841
Median	0.935	0.853	0.837	0.878	0.841

Group 0, newly identified acute to subacute infarction; group 1, existing acute to subacute infarction or old infarction; group 2, without infarction. Macrosensitivity is the average sensitivity for all groups. The model in the 14th training session exhibited the highest macrosensitivity and accuracy

### Performance of the fine-tuned LLM on the test dataset

Table 3 presents the confusion matrices of the fine-tuned model. On the test dataset, the number of group 0 was 79, of which 49 were true positives and 30 were false positives. The precision and false discovery rate of groups 0 were 0.620 and 0.380, respectively. Table 4 shows the sensitivity and accuracy data for the best fine-tuned model. The sensitivities of groups 0, 1, and 2 of the best fine-tuned model were 0.891, 0.905, and 0.959, respectively. The macrosensitivity and accuracy of the best fine-tuned model were 0.918 and 0.923, respectively. No statistically significant difference in sensitivity and accuracy was observed between the CT and MRI reports.

**Table 3:** Confusion matrices of the best fine-tuned model in the test dataset

		Reference		
Modality	Prediction	Group 0	Group 1	Group 2
All	n	55	486	293
	Group 0	49	28	2
	Group 1	5	440	10
	Group 2	1	18	281
CT	n	16	241	156
	Group 0	13	15	1
	Group 1	3	215	6
	Group 2	0	11	149
MRI	n	39	245	137
	Group 0	36	13	1
	Group 1	2	225	4
	Group 2	1	7	132

Group 0, newly identified acute to subacute infarction; group 1, existing acute to subacute infarction or old infarction; group 2, without infarction

**Table 4:** Performance of the best fine-tuned model in the test dataset

	Sensitivity				Accuracy
	Group 0	Group 1	Group 2	Macrosensitivity	
All	0.891	0.905	0.959	0.918	0.923
CT	0.813	0.892	0.955	0.887	0.913
MRI	0.923	0.918	0.964	0.935	0.933
*P*-values	0.342	0.355	0.776	0.262	0.299

The sensitivity for each group and the accuracy of CT were compared with those of MRI using Fisher’s exact test. The macrosensitivity of CT was compared with that of MRI using paired *t*-test

**Fig. 2:**
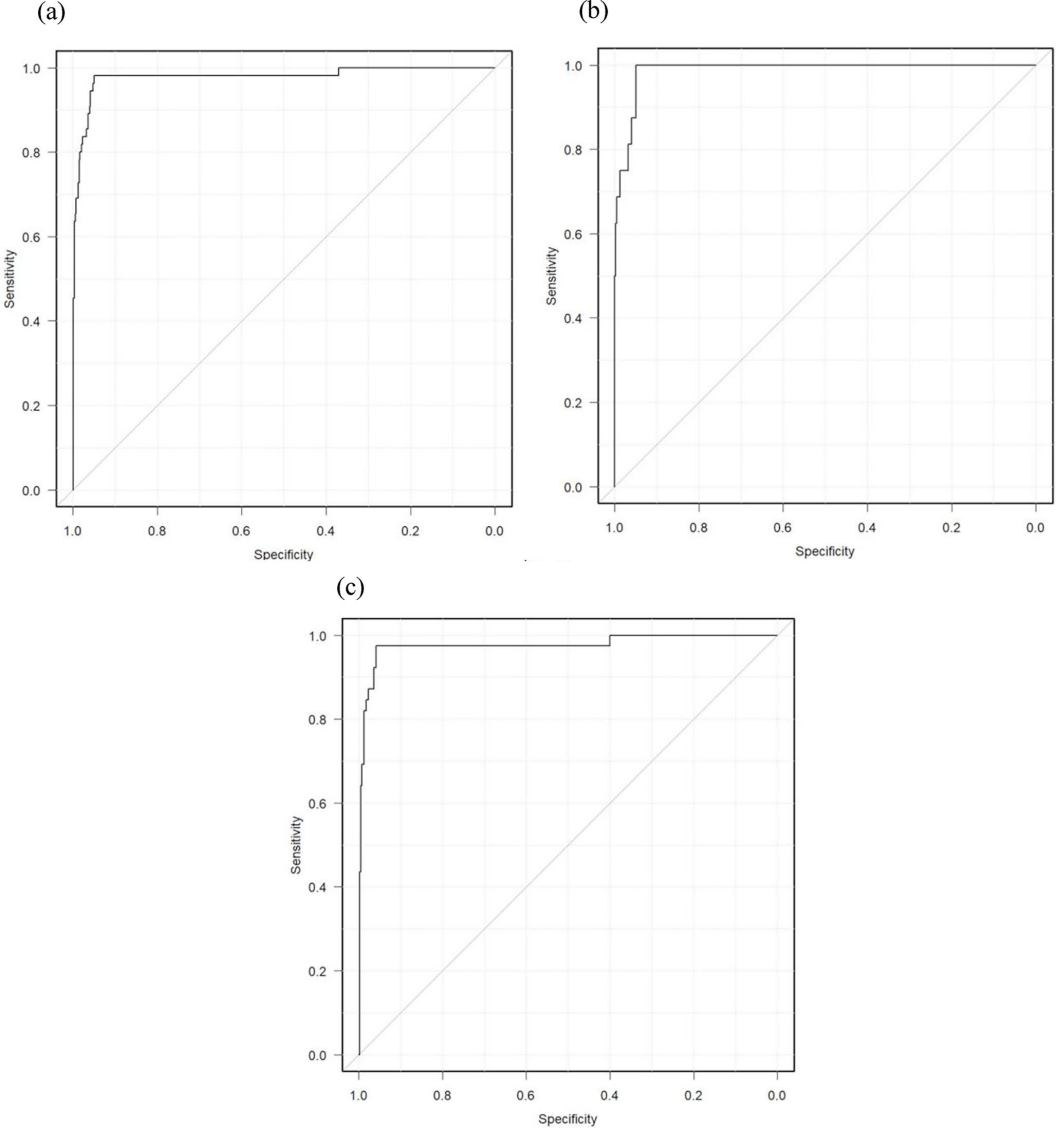
Receiver operating characteristic curve analysis for discriminating group 0 from groups 1 and 2 using the fine-tuned model in the test dataset. The area under the receiver operating characteristic curve values were 0.979, 0.988, and 0.975 for all data (**a**), CT (**b**), and MRI (**c**), respectively

The model’s performance in discriminating group 0 from groups 1 and 2 for all data, CT data, and MRI data was high, with AUCs of 0.979 (95% confidence interval [CI], 0.956–1.000), 0.988 (95% CI, 0.977–0.999), and 0.975 (95% CI, 0.945–1.000), respectively (Fig. 2).

The average time required to make a prediction was 0.115 ± 0.037 s per patient.

## Discussion

This study evaluated the feasibility of a fine-tuned LLM for classifying patients into three categories—newly identified acute to subacute ischemic stroke, known existing acute to subacute infarction or old infarction, and nonstroke—based on unstructured CT and MRI reports. The optimized fine-tuned model exhibited high performance in stroke classification.

A BERT-based fine-tuned model is well suited for integrating NLP into radiology for several reasons. First, it can be efficiently fine-tuned using publicly available pretrained models with minimal GPU resources and short training times, thereby eliminating the need for complex rule-setting or extensive programming. Second, unlike ChatGPT, which requires data to be uploaded to external servers, this model ensures privacy and security—critical considerations in the medical field—by allowing local fine-tuning [19].

Previous studies have reported the high performance of Japanese BERT models in identifying clinically significant radiology reports [11, 16, 18]. For example, Kanzawa et al. reported that brain MRI reports could be accurately classified into three categories: no brain tumor, posttreatment brain tumor, and pretreatment brain tumor [17]. Our results are consistent with those of previous studies and reinforce the potential of NLP models to improve the efficiency of radiology report processing. These results suggest that our model has promising clinical applications. It can be applied following the extraction of radiology reports containing the term “cerebral infarction” and integrating it into a medical image management and processing systems would further facilitate its use in routine practice. It could serve as the basis for an alert system that promptly notifies clinicians of patients with acute ischemic stroke requiring immediate intervention and subacute brain infarcts who also requires appropriate clinical management [4]. Furthermore, the model can streamline patient selection for large-scale research studies, including deep learning and machine learning applications, making it a valuable tool for clinical and research settings.

Class imbalance causes sensitivity discrepancies between different groups [20]. Common approaches to address this problem include undersampling and oversampling. However, oversampling carries the risk of overfitting, whereby the model becomes overly specialized in the minority class and loses generalizability to unseen data. In this study, oversampling was not feasible because of the extreme imbalance in the dataset, with group 0 (*n* = 308) being significantly smaller than groups 1 (*n* = 2,359) and 2 (*n* = 2,906). Therefore, an undersampling approach was used, adjusting the number of cases in groups 1 and 2 to *n* = 500 each. With this approach, the performance of differentiating group 0 from groups 1 and 2 achieved a high value (AUC of 0.979). In addition to sensitivity and accuracy, the precision and the false discovery rate are another important metrics for evaluating the practical performance of a clinical decision-support system. In our test set, the model generated 79 alerts for possible acute or subacute infarction, of which 49 were true positives and 30 were false positives, yielding a precision of 0.620 and a false discovery rate of 0.380. This may be due to the relatively low prevalence of acute or subacute infarction. This relatively low precision may impact clinical practice by increasing physicians’ workload, causing alarm fatigue. Further learning that increases precision while maintaining sensitivity would strengthen the system’s usefulness as an early warning tool and increase its potential utility.

This study has several limitations. First, there was an imbalance in the number of patients between the groups. However, we managed to address this problem by undersampling the data for groups 1 and 2 in the training dataset to balance the sensitivity between the groups. Second, our model was developed and evaluated using data exclusively from our institution, and its performance on external datasets is unknown. According to Walston et al., although random splitting, cross-validation, and leave-one-out methods are categorized as internal validation, the use of temporal and geographic sets is regarded as external validation [21]. In this study, a temporally external dataset was used to enhance the robustness of the results. Third, this study focused only on stroke classification and did not evaluate the model’s ability to identify other pathologies. Further research is required to evaluate its performance in detecting other conditions. Fourth, we selected the target cases based on keyword search, specifically those containing the term “cerebral infarction.” The present results reflect the performance after applying a language model following the keyword search, and therefore, both processes—keyword filtering and model-based analysis—are necessary. If all cases were included without keyword filtering, there is a risk that the reported performance could not be maintained. However, keyword search itself is a simple and accurate procedure that can be implemented easily at any facility, and thus we believe it does not pose a significant barrier to applying our results in clinical practice. Finally, a Japanese BERT model was used, and the model performance may vary in different languages. Therefore, whether our results are directly applicable to other languages remains unclear.

In conclusion, this study demonstrated that a fine-tuned LLM achieves high performance in classifying patients with newly identified acute to subacute stroke from unstructured radiology reports. Implementation of this approach in clinical practice could help prevent overlooked cases of acute to subacute ischemic stroke and improve the efficiency of case selection for research.

## Data Availability

My manuscript has no associated data or the data will not be deposited.
